# Implications of Progestin-Primed Ovarian Stimulation (PPOS) in a Patient With Diminished Ovarian Reserve (DOR) and Its In Vitro Fertilization (IVF) Outcome

**DOI:** 10.7759/cureus.54743

**Published:** 2024-02-23

**Authors:** Sanket Mahajan, Akash More, Shilpa Dutta, Jarul Shrivastava, Neha Nawale, Namrata Choudhary

**Affiliations:** 1 Clinical Embryology, Datta Meghe Institute of Higher Education and Research, Wardha, IND; 2 Clinical embryology, Datta Meghe Institute of Higher Education and Research, Wardha, IND; 3 Clinical Embryology, Datta Meghe Institute Of Higher Education And Research, Wardha, IND

**Keywords:** intracytoplasmic sperm injection (icsi), gonadotropin-releasing hormone (gnrh) antagonist, secondary infertility, diminished ovarian syndrome, progestin-prime ovarian stimulation, assisted reproductive technology

## Abstract

In this case study, a 39-year-old woman pursuing treatment for secondary infertility at our infertility clinic was visited by her 42-year-old husband. The couple had a history of failed attempts, including two intrauterine insemination (IUI), two intracytoplasmic sperm injection (ICSI) cycles, and two miscarriages. Diminished ovarian reserve (DOR) was noted in the patient's medical profile. A gonadotropin-releasing hormone (GnRH) antagonist, cetrorelix acetate, was given to the patient at a daily dosage of 0.25 mg to treat their condition once the maturing follicle had grown to a diameter of 10 mm.

Following the administration of the GnRH antagonist, the first oocyte pick-up (OPU) procedure was conducted. During this process, two oocytes were successfully retrieved. Subsequently, ICSI was performed to facilitate fertilization. However, during the fertilization check, it was observed that no pronuclear fertilization (PN) formations occurred, leading to a cessation of development. Following the initial failure, an ovarian stimulation strategy based on progestin priming was implemented. Progestin is administered using this technique to ready the endometrium for the implantation of the embryo. After the modified ovarian stimulation protocol, an additional beta-human chorionic gonadotropin (β-hCG) test was verified as a successful clinical pregnancy outcome.

## Introduction

One of the most common human issues, infertility, has long had negative effects on the psychological and social well-being of families or couples. Approximately 10% to 15% of couples worldwide are affected by infertility, which has been on the rise in recent decades [1]. Diminished ovarian reserve (DOR) is one of the main causes of infertility [2]. According to certain research, infertile women with DOR experienced poor ovarian response during in vitro fertilization, higher miscarriage rates, a lower chance of having at least one euploid blastocyst, and an increased risk of cycle cancellation [3]. Due to the negative side effects of gonadotropin-releasing hormone (GnRH) analogs, progestins gained increasing attention in research in recent years as a potential replacement for GnRH analogs in managing the luteinizing hormone (LH) surge. Previously, it was believed that progestin could serve as a substitute medication to suppress the early LH surge during control ovarian stimulation (COS). In some studies, endogenous progesterone may prevent the rise in LH if there is no spontaneous surge during the luteal phase, particularly in the context of COS [4].

Without causing ovarian hyperstimulation syndrome, progestin is used to prevent the estrogen-induced LH surge. Patients with normal reserve and polycystic ovarian syndrome, among other ovarian conditions, have successfully used the progestin protocol [5]. It is unknown if progestin-primed ovarian stimulation (PPOS) can also be used as a substitute for conventional in vitro fertilization (IVF) treatments, such as mild stimulation for older women with DOR [6]. In the past 10 years, new protocols, such as GnRH antagonist protocols and mild stimulation protocols, have been proposed in response to the ongoing need for ovarian stimulation protocols with better efficacy, safety profile, and user convenience. One of these new ovarian stimulation protocols is PPOS, which uses progestin along with gonadotrophin and triggers ovulation with a low dose of human chorionic gonadotropin (hCG) and a GnRH agonist [7]. This case study highlights the utilization of PPOS therapy to treat patients with DOR, thereby improving the chances of positive clinical pregnancy.

## Case presentation

Patient information

This case study is based on a middle-aged couple who visited our IVF center located in Maharashtra, India. In their quest to fulfill the dream of pregnancy, a 39-year-old middle-aged woman was experiencing secondary infertility, attributed to previous abortions within four of the 10 years of married life. The couple was counseled regarding the procedure, and duly informed consent was obtained from them. The male partner was 42 years old. Both partners had no history of drinking, smoking, or any tobacco addictions.

Medical/surgical history

The case study revolves around a female who had a medical history of dengue and two abortions in her married life. Additionally, she experienced two failed intrauterine insemination (IUI) and two intracytoplasmic sperm injection (ICSI) cycles at a previous fertility center. They had no sexual problems in their marriage but faced secondary infertility for four years within their 10 years of marriage life. Asthma, tuberculosis, and hypertension were absent in both. The male partner had a history of hypotension for the last two years and had been taking midodrine hydrochloride tablets orally, 2.5 mg once a day, for the first time. This was their initial consultation at our clinic for the treatment of their infertility condition.

Physical examination and investigation

The female body mass index (BMI) was 23.5 kg/m^2^, and for the male, it was 24.6 kg/m^2^. To identify the underlying reason for their infertility, both couples had thorough infertility evaluations. After performing the husband's semen analysis, the sperm count was reported to be 13 million/mL, the morphology of sperms in the semen sample was 97% defective, the normal morphology of sperms in semen was 3%, and progressive motility was 65%. According to his report, his semen profile indicated oligozoospermic. Table 1 shows the semen reports of the patient observed during the semen analysis.

**Table 1:** Semen parameter of the male partner. WHO, World Health Organization

Parameter	Observed limit	Reference limit (WHO 2021)
Semen volume	1.4 mL	>1.4 mL [8]
Morphological defects	97%	96% [8]
Normal morphology	3%	>4 % [8]
Vitality	45%	>54% [8]
Progressive motility	31%	>30% [8]
Count	13 million/mL	16 million/mL [8]
pH	7.1	>7.2 [8]
Color	Opaque white	Opaque white [8]
Viscosity	Liquified	

Upon examination of the report, it was inferred that the patient had a DOR. Her hormonal levels were not in the normal range. In addition, there were no confounding factors observed. The anti-Müllerian hormone (AMH) level was 0.78 ng/mL, and the follicle-stimulating hormone (FSH) level was 17 IU/L. The patient had a low AMH and a high level of FSH, as mentioned in Table 2.

**Table 2:** Hormonal investigations of the female partner. AMH, anti-Müllerian hormone; FSH, follicle-stimulating hormone; LH, luteinizing hormone

Hormonal profile	Patient value	Reference value
AMH	0.78 ng/mL	0.8-1.0 ng/mL [9]
FSH	17 IU/L	10 IU/L [9]
LH	7.0 U/L	5 U/L [10]

Treatment

**Figure 1:**
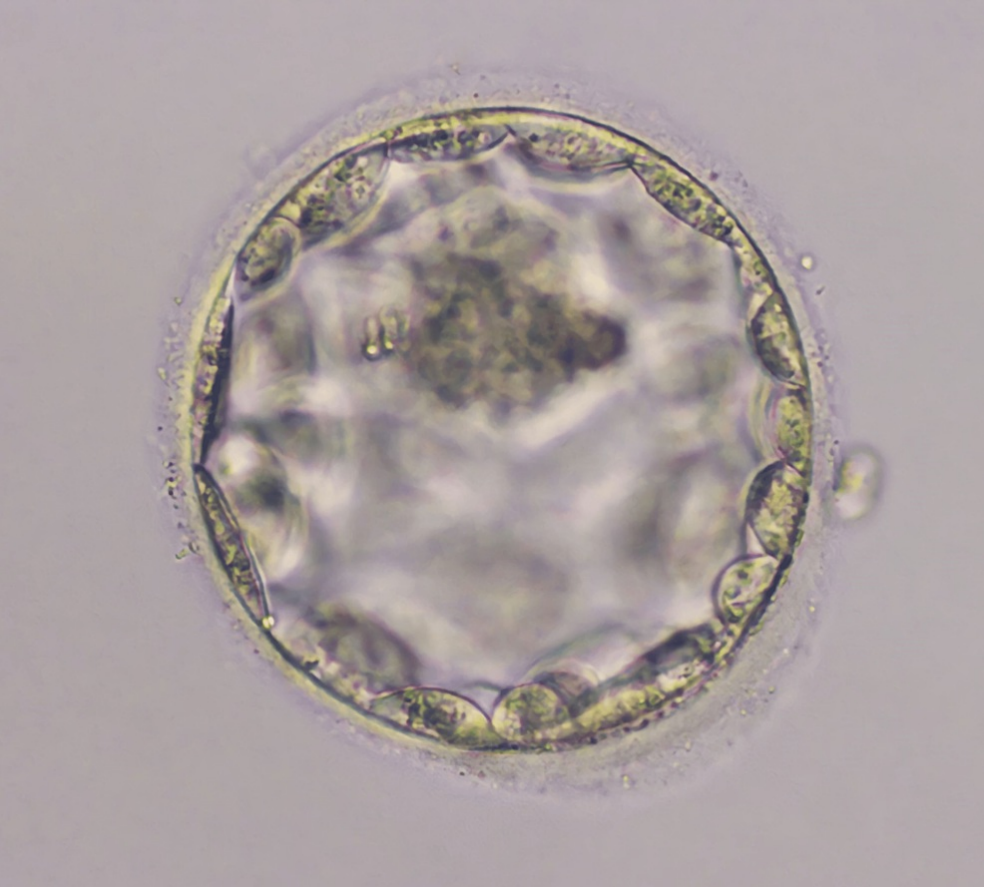
Day 5 blastocyst (4AA quality) transferred to the patient.

The use of cetrorelix acetate, a GnRH antagonist, is an essential part of assisted reproductive technology (ART) treatment. The patient was prescribed a cetrorelix acetate dose of 0.25 mg twice a day on day 5 and once on day 6 to address maturing follicles that had grown to a diameter of 10 mm. The patient was recommended for the first oocyte retrieval procedure, and the administration of the GnRH antagonist commenced on day 2. On the same day, we administered a subcutaneous injection of 10,000 IU of hCG to induce the maturation of oocytes. The patient was triggered for oocyte pick-up (OPU) on day 14, and the OPU was scheduled 36 hours later. Two oocytes were retrieved during the procedure, and on the same day, ICSI was performed. However, during the fertilization check, it was observed that no pronuclear fertilization (PN) occurred, leading to a stop in embryo growth. Later, upon examining the blood test report, it showed a low level of AMH, leading to DOR. Subsequently, the administration of 10 mg dydrogesterone orally, twice a day, from day 5 to day 25 of the menstrual cycle was initiated. This treatment was crucial for hormonal support during a specific menstrual phase cycle. After completing the treatment, the patient was again suggested for a second OPU, during which the patient was triggered for OPU 36 hours before the procedure. During OPU, 11 oocytes were retrieved, comprising three mature oocytes (MII), five metaphase I oocytes (MI), and three germinal vesicle oocytes (GV). On the same day, ICSI was performed, leading to the initiation of fertilization for the embryos. On day 5, the embryo transfer was scheduled, and one Day 5 embryo was transferred. Good-quality blastocyst (4AA) was transferred, and the patient had no complications during the procedure. Figure 1 shows the blastocyst transferred to the patient.

Follow-up

After the embryo transfer procedure, the patient was advised to take prescription medications, such as 200 mcg of oral progesterone, for the next 14 days to support the growth of the uterine lining for better implantation. Frequent follow-up appointments enabled the patient to be closely observed while her progress was assessed. The patient also received lifestyle modification advice, which included recommendations for a healthy diet, frequent exercise, and avoiding potential risks. Throughout the follow-up phase, sufficient support and guidance were given to address any concerns or inquiries that came up. The likelihood of a successful outcome was increased by closely monitoring the patient's overall health and the progress of the pregnancy. The patient's pregnancy was verified by a positive human chorionic gonadotropin (β-hCG) test two weeks later at scheduled follow-up appointments. The reported value of β-hCG was 1,150 mIU/mL.

## Discussion

Kuang et al., in 2015, compared the pregnancy outcomes of women undergoing IVF/ICSI with frozen embryo transfer. They found that this protocol was superior to the short protocol in preventing premature LH surges and equally effective in improving IVF/ICSI outcomes. Dydrogesterone (DYG), progesterone capsules (PCs), and medroxyprogesterone acetate (MPA) are the three main exogenous progesterone administration methods used in PPOS [4].

In 2019, a domestic meta-analysis included 2,270 cycles in the PPOS group and 2,463 cycles in the microstimulation group. The findings showed that compared to the microstimulation protocol, the PPOS protocol produced a higher rate of high-quality embryos and a lower rate of cycle cancellation for patients with DOR [11]. We tried PPOS treatment on a single patient, and the pregnancy was achieved in a patient with DOR.

Additionally, prior research has indicated that there are no unfavorable effects, such as low cycle cancellation rates or miscarriages, upon the administration of the PPOS protocol [12]. Therefore, a different meta-analysis was unable to show a difference in the live birth rate between protocols utilizing PPOS and GnRH antagonists [13]. Accordingly, a different meta-analysis was unable to demonstrate a difference in the live birth rate between protocols utilizing PPOS and GnRH antagonists [14]. Our case report is in alignment with the previous research that PPOS may be an effective alternative therapy for patients having DOR and poor-quality oocytes. However, since this case report is performed on one patient, further randomized trials are anticipated to validate the result of this study.

## Conclusions

This case report highlights an important alternative stance that can be taken for patients with poor oocyte quality resulting from DOR. PPOS therapy may serve as a cost-friendly and effective option to answer the specific epithet. However, further random trials are anticipated for proper validation of the result and to make it a standard protocol in these scenarios to enhance the rate of successful pregnancy.

## References

[1] Zargar M, Pourjafar Z, Salemi S, Barati M (2022). Evaluation of the consequences of double ovarian stimulation through shanghai protocol in patients with poor ovarian respond. Res Sq.

[2] Carson SA, Kallen AN (2021). Diagnosis and management of infertility: a review. JAMA.

[3] Zhang W, Zhang L, Liu Y (2021). Higher chromosomal aberration frequency in products of conception from women older than 32 years old with diminished ovarian reserve undergoing IVF/ICSI. Aging (Albany NY).

[4] Kuang Y, Chen Q, Fu Y (2015). Medroxyprogesterone acetate is an effective oral alternative for preventing premature luteinizing hormone surges in women undergoing controlled ovarian hyperstimulation for in vitro fertilization. Fertil Steril.

[5] Yu CM, Dai XL, Wang YF, Gao TT, Cao F, Xia XY, Chen L (2019). Progestin-primed ovarian stimulation improves the outcomes of IVF/ICSI cycles in infertile women with diminished ovarian reserve. J Chin Med Assoc.

[6] Tu X, You B, Jing M, Lin C, Zhang R (2021). Progestin-primed ovarian stimulation versus mild stimulation protocol in advanced age women with diminished ovarian reserve undergoing their first in vitro fertilization cycle: a retrospective cohort study. Front Endocrinol (Lausanne).

[7] Xi Q, Tao Y, Qiu M, Wang Y, Kuang Y (2020). Comparison between PPOS and GnRHa-long protocol in clinical outcome with the first IVF/ICSI cycle: a randomized clinical trial. Clin Epidemiol.

[8] Boitrelle F, Shah R, Saleh R (2021). The sixth edition of the WHO manual for human semen analysis: a critical review and SWOT analysis. Life (Basel).

[9] Wang S, Zhang Y, Mensah V, Huber WJ 3rd, Huang YT, Alvero R (2018). Discordant anti-müllerian hormone (AMH) and follicle stimulating hormone (FSH) among women undergoing in vitro fertilization (IVF): which one is the better predictor for live birth?. J Ovarian Res.

[10] Zhang M, Sun J, Wang Y (2023). The value of luteinizing hormone basal values and sex hormone-binding globulin for early diagnosis of rapidly progressive central precocious puberty. Front Endocrinol (Lausanne).

[11] Li J, Li Y, Li M (2023). Analysis of cumulative live birth rate outcomes of three ovarian stimulation protocols in patients after laparoscopic cystectomy of ovarial endometrioma: a retrospective cohort study. Reprod Health.

[12] Guan S, Feng Y, Huang Y, Huang J (2021). Progestin-primed ovarian stimulation protocol for patients in assisted reproductive technology: a meta-analysis of randomized controlled trials. Front Endocrinol (Lausanne).

[13] Alexandru P, Cekic SG, Yildiz S, Turkgeldi E, Ata B (2020). Progestins versus GnRH analogues for pituitary suppression during ovarian stimulation for assisted reproductive technology: a systematic review and meta-analysis. Reprod Biomed Online.

[14] Filippi F, Reschini M, Polledri E (2023). Progestin-primed ovarian stimulation for fertility preservation in women with cancer: a comparative study. PLoS One.

